# Investigating assessment standards and fixed passing marks in dental undergraduate finals: a mixed-methods approach

**DOI:** 10.1186/s12909-025-06944-y

**Published:** 2025-04-03

**Authors:** Ting Khee Ho, Lucy O’Malley, Reza Vahid Roudsari

**Affiliations:** 1 Division of Dentistry, School of Medical Sciences, Faculty of Biology, Medicine and Health, Manchester Academic Health Science Centre, University of Manchester, Oxford Road, Manchester, M13 9PL UK; 2 Department of Restorative Dentistry, Faculty of Dentistry, Universiti Kebangsaan Malaysia, Jalan Raja Muda Abdul Aziz, Kuala Lumpur, 50300 Malaysia

**Keywords:** Education, Dental, Education measurement, Standard setting, Mixed-methods

## Abstract

**Background:**

Standard setting is widely practised in healthcare education programmes and specialty examinations in many countries. However, Malaysian dental institutions still arbitrarily set a fixed 50% pass-fail assessment threshold. The purpose of this mixed-methods study was to explore faculty members’ experiences and practices in student assessment, their perceptions of the assessment standards employed by the faculty, and their views on the fixed passing standard of 50% in the dental undergraduate final professional examination.

**Methods:**

A mixed-methods study was conducted at a single dental school in Malaysia. An online questionnaire was administered to eligible lecturers, followed by in-depth interviews with volunteer respondents. Quantitative data were analysed descriptively using the statistical software Jamovi; qualitative data was analysed with inductive thematic analysis process in Microsoft Excel.

**Results:**

A total of 26 lecturers responded to the questionnaire (55% response rate), and 12 of these respondents also completed interviews. All respondents had experience in writing and developing assessments for students and reported that post-hoc assessment analysis and standard setting were not routinely carried out. The questionnaire analysis revealed that 13 respondents (50%) felt that the passing marks for the final exam were fair, 9(34.6%) were neutral, and 4(15.4%) strongly disagreed/disagreed. Four themes emerged from the qualitative data: (1) Trust in the institutional quality assurance processes (2) Reflections on the passing mark as passing standard (3) Potential barriers to standard setting (4) Future faculty development strategies.

**Conclusion:**

Arbitrary passing marks are common practise in dental education in this region. Our research revealed mixed confidence among participants in using an arbitrary fixed passing marks to make pass-fail decisions for dental high-stakes examinations. Low level of exposure and knowledge about educational measurement has restricted the application of post-hoc assessment analysis and standard-setting practices at the institute. Most participants were positive about exploring and learning methods to improve assessment practices and ensure fair passing standards. Any implementation of standard setting in similar contexts will need careful thought around training, support and infrastructure.

**Supplementary Information:**

The online version contains supplementary material available at 10.1186/s12909-025-06944-y.

## Background

Assessment is a crucial component of any undergraduate dental programme. It helps measure students’ progress throughout the curriculum and decide their readiness for graduation at the exit point. It is essential that dental graduates who pass such exams have demonstrated the minimum standards set by their national statutory body [1–3].

National statutory bodies are legally recognised organisations that regulate and oversee professions within a country [4]. Their function includes licensure or certifications for clinical practice, ensuring practitioners meet educational and professional standards, and enforcing standards for education, training, and conduct. They also accredit educational programmes to ensure adequate training and provide advisory services to government bodies, stakeholders, and the public on professional issues.

In Malaysia, the Malaysia Dental Council (MDC) serves as the statutory professional body responsible for regulating and overseeing dental practice [5]. To ensure the quality of the dental graduates, the dental assessment must comply with MDC standards while also adhering to the academic standards and guidelines of quality assurance agencies such as the Malaysian Qualifications Framework (MQF) and the Code of Practice for Programme Accreditation (COPPA) [6]. The MDC has developed a standard document that sets out the criteria and competencies required of fresh graduates from dental bachelor’s degree programmes in Malaysia [2]. The final-year examination in Malaysian dental schools must demonstrate students’ attainment of the clinical competencies set by the MDC, as the dental degree will lead to registration with the MDC. These licensing and certification tests must be reliable, consistent, and valid to ensure stakeholders can trust the value of the qualification awarded to dental students [7].

The pass-fail decision of an examination is based on the passing mark, also known as the pass mark, cut-score or minimum achievement level for an examination. Traditionally, this has been set arbitrarily at a predetermined fixed percentage [8]. The concept of criterion-based standard setting slowly gained attention among educators in the 1970s when the standard set was based on mastery models to make pass-fail decisions [9]. In a professional and regulatory context, the term “standard” can refer to the expected level of proficiency a professional must achieve. Standard setting, also referred to as setting performance standards, is the process of establishing a conceptual measurement for the level of quality or ‘minimum adequate level’. This then determines the minimal level of skill and knowledge required and translates the conceptual definition of competence into an examination passing score; this then discriminates between the students who pass and those who fail [8, 10].

There are two primary categories of standard setting methods: relative methods and absolute methods [11, 12]. The relative standard is established by comparing the performance of an individual against other examinees within the same cohort. The standard set is based on a certain percentage of examinees who will achieve a passing outcome. It is often used to rank the position of examinees, particularly in situations like admission selection for educational programmes where the available positions are limited [12, 13]. Absolute standard setting methods also known as criterion-referenced, involve reviews by experts referred to as *judges* or *panellists*. These judges or panellists go through the test items or examinees’ performance data to make decisions in determining the minimally acceptable level for the examination [14]. The absolute standard setting method is preferred in credentialing and certification examination as the decision to be made is based on the minimally acceptable level of an individual’s safety as a practitioner rather than merely comparing their safety to peers [15].

More recently, the application of standard setting in healthcare education programmes has been widely practised and implemented in bachelor’s degrees and speciality examinations in many countries [1, 15–21]. Among the various methods, the Angoff and modified Angoff methods were the most commonly used, with the choice of standard setting method often depending on the types of item formats used in the assessments [20, 21]. Universities in the United Kingdom (UK) typically employ a common grading system, designing 40% as the pass mark for most courses [22]. However, the UK’s General Dental Council (GDC) and General Medical Council (GMC) have explicitly stated in their respective standards documents that the expected performance level for students in each assessed area must be fair and aligned with curriculum outcomes. Additionally, an appropriate standard setting process must be employed for summative assessments to ensure that the standards are stable from year to year [1, 23]. To comply with the university guidelines, Tekian and Norcini recommended test equating method to rescale the new passing score as a result of standard setting practice to the university’s policy-determined fixed passing mark [24].

Research has shown that after applying absolute standard setting methods to retrospective cohorts, the passing marks and passing rates have changed significantly. For example, in one study, the predetermined passing mark for a final-year dental examination was 50%, but this increased to 54.6% after applying the modified Angoff method, resulting in the passing rate dropped from 100 to 80% [25]. Similarly, using modified Angoff and Bookmark methods lowered the passing rate of an Advanced Practice Nurse certification exam from 93.4 to 52.9% and 57%, respectively, compared to a fixed passing mark of 60% [26]. These findings highlight concerns about the validity and reliability of graduates’ competency levels and the potential risk of incorrect pass-fail decisions.

Regulators require dental schools to demonstrate that their graduates have the required qualities and have attained the requisite clinical competency, thereby being accountable to stakeholders. In Malaysia, although the practice of standard setting in healthcare education has been reported [25, 27, 28], most dental institutions continue to use an arbitrary fixed passing mark of 50% for their final-year dental professional examinations as per university policy. At present, there is lack of literature on how faculty measure and determine assessment standards to align with the 50% passing mark. The assessment experience and dental academics’ confidence in the 50% passing mark are essential for evaluating the need for examination system improvements. The purpose of this mixed-methods study was to explore faculty members’ experiences and practices in student assessment, their perceptions of the assessment standards employed by the faculty, and their views on the fixed passing standard of 50% in the dental undergraduate final professional examination.

## Methods

### Study design and setting

**Fig. 1:**
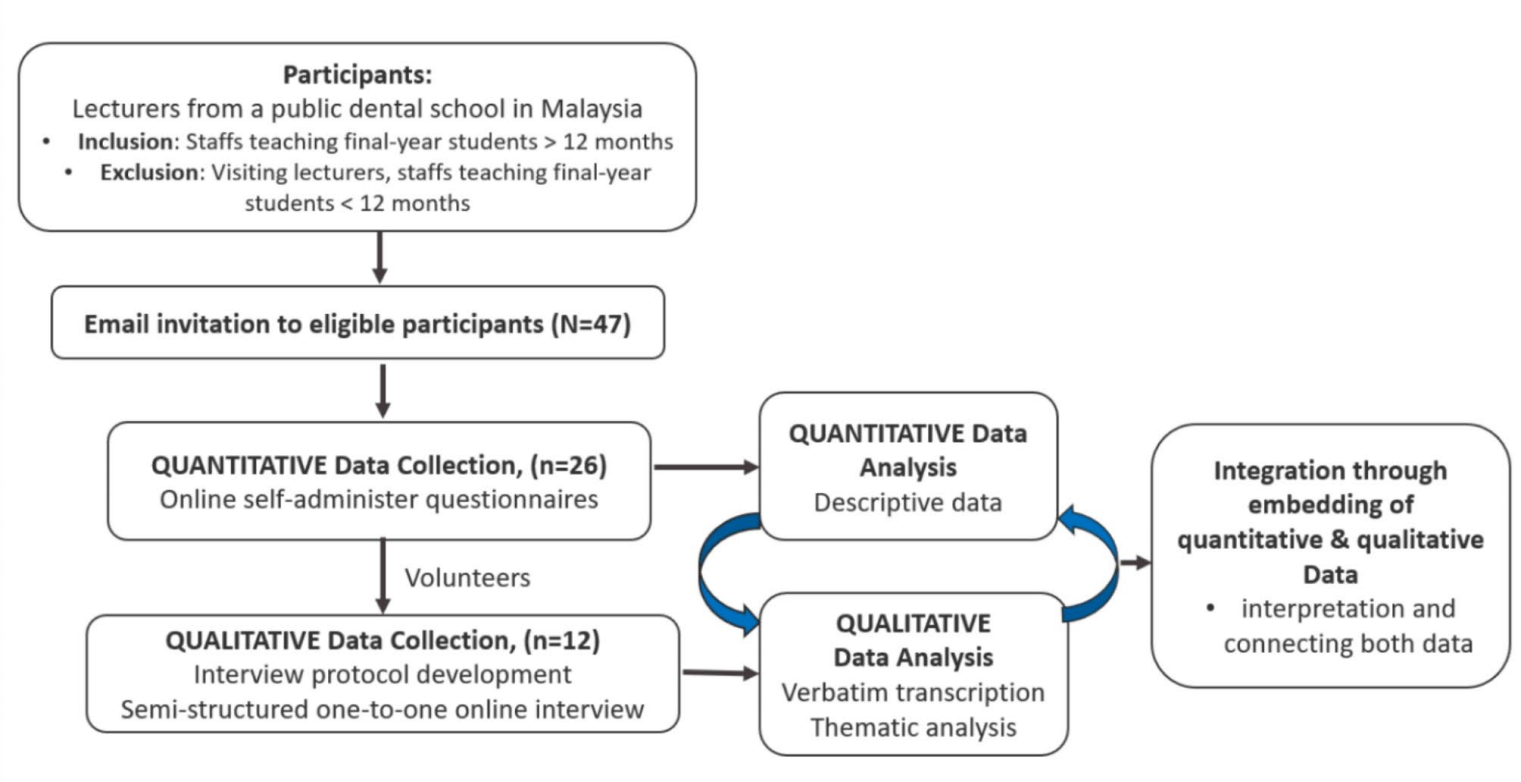
Diagram showing study design and protocol: Mixed-method approach

This study employed an explanatory sequential mixed methods design, consisting of a quantitative phase followed by a qualitative phase [29]. The quantitative data explored experiences of and practices in assessment management as well as perceptions of setting standards in assessment. The qualitative element of this study delved into the perceptions of standard setting more deeply. The quantitative data were collected through questionnaires, and findings were used to develop the qualitative interview questions. Qualitative data were gathered through semi-structured interviews conducted with a smaller sample of participants (Fig. 1). The anonymous self-administered questionnaires were developed in Qualtrics XM (Qualtrics, Provo, UT), and the one-to-one interviews were carried out virtually using the Zoom online conferencing platform (Zoom Video Communications, San Jose, CA). All data collection took place virtually between June 2023 to August 2023. The first author, a staff member at the same dental institution as the participants, had no teaching or administrative responsibilities during the research period. Participants were reassured that the interview data would be processed confidentially and would not influence their professional careers.

### Study participants and sampling

Criterion sampling was used to recruit all dental lecturers with more than 12 months of teaching experience across various disciplines involving final-year undergraduate dental students at a single public university in Malaysia. Visiting and part-time lecturers were excluded from the survey due to their lack of involvement in curriculum development and examination board committee meetings. Based on information from the faculty’s administrative office, a total of 47 eligible staff members were identified who could participate in this study.

An invitation email containing a link to an online questionnaire was sent to all eligible participants with three follow-up email reminders. Upon completing the questionnaire, the participants were invited to attend one-to-one interviews with an online link to register their interest. The separate link to register for the interviews ensured the anonymity of the survey responses. Subsequent appointments for virtual one-to-one interviews were arranged at times convenient for the participants. As a token of appreciation for their time, stationery souvenirs were prepared for the interview participants. The final sample size for the interviews was limited to the number of participants who volunteered and determined when data saturation was reached with no new data emerging.

### Quantitative questionnaire study

To collect the quantitative data, a questionnaire was developed by authors using Qualtrics XM. It consisted of four sections: (1) staff’s teaching background; (2) their experience and practice in assessment including analysing performance data in the examination; (3) their perception on analysing performance data; and (4) their perception towards setting passing marks for final examinations. Three content experts were contacted to evaluate the face and content validities of the questionnaire. They were asked for the appropriateness and clarity of items for the respective constructs by answering the expert validation form [30, 31]. The expert feedback included free-text comments on suggestions for survey improvement. All expert reviewers were experienced health professional educators with expertise in assessment and social science-based research. Based on the feedback given by the expert reviewers, the questionnaire was revised to improve relevancy and clarity. The online questionnaire was piloted on three academics who shared a teaching portfolio similar to the intended sample. This pilot aimed to assess user readability, clarity, comprehensibility, and the time needed to complete the questionnaire. The items were amended according to the comments and feedback after the validation process to improve overall clarity for the participants.

The final questionnaire consisted of a total of 20 items. The items used in the study are explained in Supplementary File 1. Regarding teaching background, the questionnaire collected information on the number of years spent teaching and their main teaching discipline. Additionally, nine items inquired about their experience in assessment management and analysing performance data of assessment, five items explored the staff’s perception on analysing performance data, and four items related to their perception about setting passing standards in the final professional examination. The questionnaire included open-ended questions to explore perceptions of fixed passing marks; these informed the questions asked in the qualitative interviews.

### Qualitative interview study

The aim of the individual in-depth interviews was to explore personal perceptions about how the passing standard is measured in the dental undergraduate final professional examinations and whether how confident staff are that this fixed passing mark reflects the minimum level of competency required for a dental graduate. Semi-structured in-depth interview guides were developed by authors in accordance with guidelines outlined by Kalio et al. (2016) and these questions were refined using the findings from the questionnaire, specifically responses to the open-ended questions in the questionnaire were used to inform the question topics for the interviews. The semi-structured interview guide was used to ensure consistency in data collection while encouraging participants to share their views (Supplementary File 2). Pilot interviews were conducted with two volunteers who had previously completed the pilot questionnaire phase. This was to assess the suitability of the interview questions and to estimate the time required for interview completion. Interviews were conducted by the main author, a PhD student with training in dental education, and quantitative and qualitative research design.

Prior to commencing the interviews, participants were informed of the purpose of the study, and informed consent was obtained. All interviews were conducted in English, with durations ranging from 45 to 60 min. These sessions were audio-recorded using the built-in recording function of the Zoom online conferencing software. The use of webcam was optional for participants; however, all participants chose to turn on their webcams. It helped to build rapport, enhance communication, and allow for eye contact during the interview. Participants were reassured that the visual data, such as video, was not recorded, as the focus was solely on capturing their verbal responses. The audio data was subsequently transcribed (intelligent verbatim) by the first author. The transcriptions were anonymised by the first author before undergoing data analysis.

### Data analysis

Statistical software Jamovi version 2.2.5 (Sydney, Australia) [32] was used for quantitative data analysis after extracting the responses from Qualtrics questionnaire form as an Excel sheet. These quantitative data were analysed descriptively. The data presentations were created graphically using Microsoft Excel spreadsheets [33].

The authors organised and analysed the open-ended survey comments and interview transcriptions using Microsoft Excel [34]. Braun and Clarke’s six-step inductive thematic analysis framework was employed to analyse the transcriptions [35]. The first author read and re-read the transcriptions and identified quotes and codes using an iterative process. The data were transferred to Microsoft Excel for storage. A table with columns was created, with the following headings: interview ID, quote, code, subtheme and theme. If a quote presented with two codes, it was duplicated to include the second code. The second and third authors double-checked the reliability and accuracy of the transcription and coding. Based on the codes, the subthemes and themes were independently generated by all authors by combining codes with similar meanings and characteristics. The subthemes and themes were compared and discussed among the researchers. Final themes, subthemes, quotes and interpretations were achieved by consensus in cases of disagreement.

### Ethical considerations

Study approval was granted jointly by the ethics committees of the University of Manchester (ref: 2023-15451-27380) and the National University of Malaysia (Universiti Kebangsaan Malaysia) (ref: JEP-2023-204) by the institutions’ Ethics Committees. Participants in the questionnaire provided implied consent (by ticking a box indicating they were happy to proceed with the questionnaire).

Interview participants provided electronic written informed consent. Participants were reassured that their involvement was entirely voluntary, their professional competence would not be judged and their insights would not impact their future career prospects. Each participant was assigned a pseudonym to be used in analysis and reporting.

## Results

### Characteristics and experience of the participants

A total of 47 eligible participants were contacted, with 26 participants (55% response rate) responding to the questionnaire. These participants had an average of 11.7 years (SD 7.3) experience in teaching final-year students (Table 1). The participants were categorised into apprentice (1–4 years), professional (5–9 years) and expert (> 10 years) based on their teaching experience, aligning with the teaching career’s life cycle model proposed by Steffy and Wolfe [36]. Out of the 26 participants, 12 volunteered for one-to-one interviews. More than two thirds of the interviewees had more than 10 years of teaching experience. The background of the participants in the study showed a wide range of sampling across various disciplines.

**Table 1:** Distribution of participants in the questionnaire and interview according to their teaching experience and discipline

	Questionnaire, *n* (%) (*n* = 26)	Interview, *n* (%) (*n* = 12)
Years of experience (Years)		
1–4 (Apprentice)	5(19.2)	3(25.0)
5–9 (Professional)	10(38.5)	1(8.3)
> 10 (Expert)	11(42.3)	8(66.7)
Discipline		
Dental Public Health	3(11.5)	1(8.3)
Endodontics	4(15.4)	1(8.3)
Oral & Maxillofacial Surgery	2(7.7)	-
Oral & Maxillofacial Radiology	1(3.8)	1(8.3)
Orthodontics	2(7.7)	1(8.3)
Paediatric Dentistry	2(7.7)	2(16.7)
Periodontics	4(15.4)	1(8.3)
Prosthodontics	3(11.5)	3(25.0)
Restorative Dentistry	5(19.2)	2(16.7)

### Quantitative data

Content validity showed a total inter-rater agreement of 83%, and the content validity index was 98.15% [37, 38]. The questionnaire responses can be seen in Table 2, measured by frequency and percentage. Overall the questionnaire showed that all lecturers were experienced assessors. The majority (*n* = 25, 96.2%) were experienced in writing examination items, including multiple choice questions (MCQs) or one-best answer (OBA) questions and all lecturers had experience in writing multiple short answer (MSA) or multiple short essay (MSE) questions. However, most did not review performance data on examination items after the examinations, such as reviewing student responses to MCQ/OBA questions (*n* = 17, 65.4%) or assessing the difficulty index, discriminatory index, and distractor efficiency in MCQ/OBA questions (*n* = 21, 80.8%). Among the respondents, only eight (30.8%) had attended training in the subject of assessment, testing, or measurement. Four (15.4%) respondents had exposure to standard setting methods in assessment, when exploring further in type of methods in dropdown choices, one respondent was familiar with Angoff; one respondent was familiar Angoff and Borderline Regression; a further two respondents reported being familiar with methods but were unable to name them.

**Table 2:** The experience and practice in assessment among participants (questionnaire data)

Activities:	Respondents (*n*)	Percentage (%)
1. Developing course assessments (collecting and compiling questions) for your students		
None	0	0
Yes	26	100
if yes, frequency in a year		
once in a year	6	24.0
more than once in a year	19	76.0
2. Writing MCQ/OBA question		
None	1	3.8
Yes	25	96.2
if yes, frequency in a year		
once in a year	3	12.0
more than once in a year	22	88.0
3. Writing multiple short answer (MSA) /multiple short essay (MSE) question		
None	0	0
Yes	26	100
if yes, frequency in a year		
once in a year	2	8.0
more than once in a year	23	92.0
4. Analysing test-takers responses to the choices in MCQ/OBA questions		
None	17	65.4
Yes	9	34.6
5. Analysing test statistics, such as difficulty index, discriminative index, and distractor efficiency in MCQ/OBA questions		
None	21	80.8
Yes	5	19.2
6. Developing large-scale educational assessments, such as at the national level, Professional Qualifying Examination (PQE)		
None	21	80.8
Yes	5	19.2
7. Setting performance standards which determine passing marks on an assessment		
None	21	80.8
Yes	5	19.2
8. Taken any training/course in assessment, testing, or measurement		
None	12	46.2
Yes	8	30.8
Cannot recall	6	23.1
9. Know any standard-setting methods used in assessment		
None	22	84.6
Yes	4	15.4

**Fig. 2:**
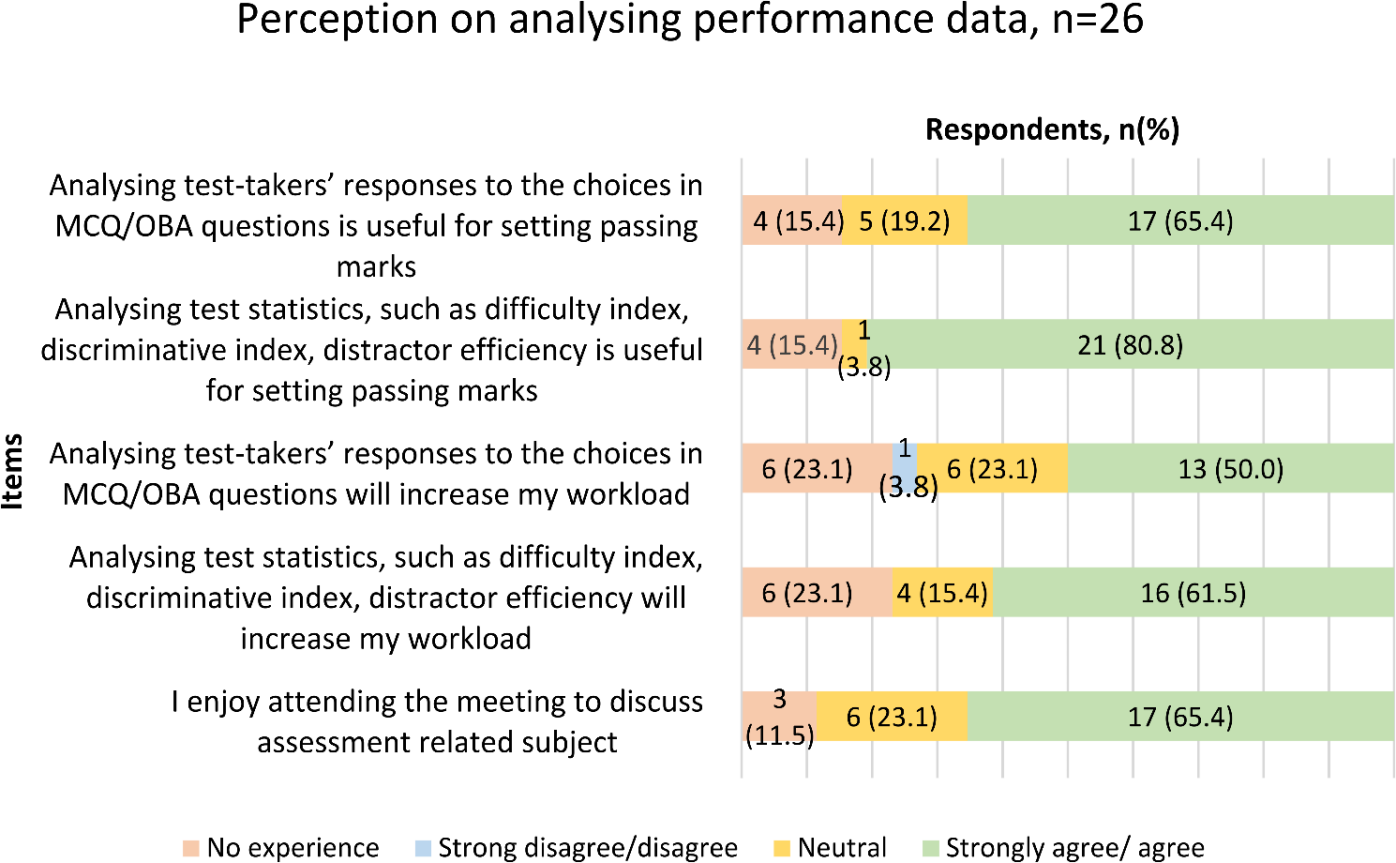
Staff’s perception in analysing performance data in the dental undergraduate final professional examination

The questionnaire also further explored the staff’s perception on the value of analysing performance data after the final examination and their opinions towards their workload (Fig. 2). The majority of respondents (65.4%) believed analysing test-takers’ responses to MCQ/OBA questions was useful for setting passing marks, and 21 respondents (80.8%) felt similarly about reviewing performance data on examination items (test statistics) after the examination. However, 50% were concerned that reviewing MCQ/OBA responses would increase their workload, and 61.5% expressed similar concerns about analysing test statistics.

**Fig. 3:**
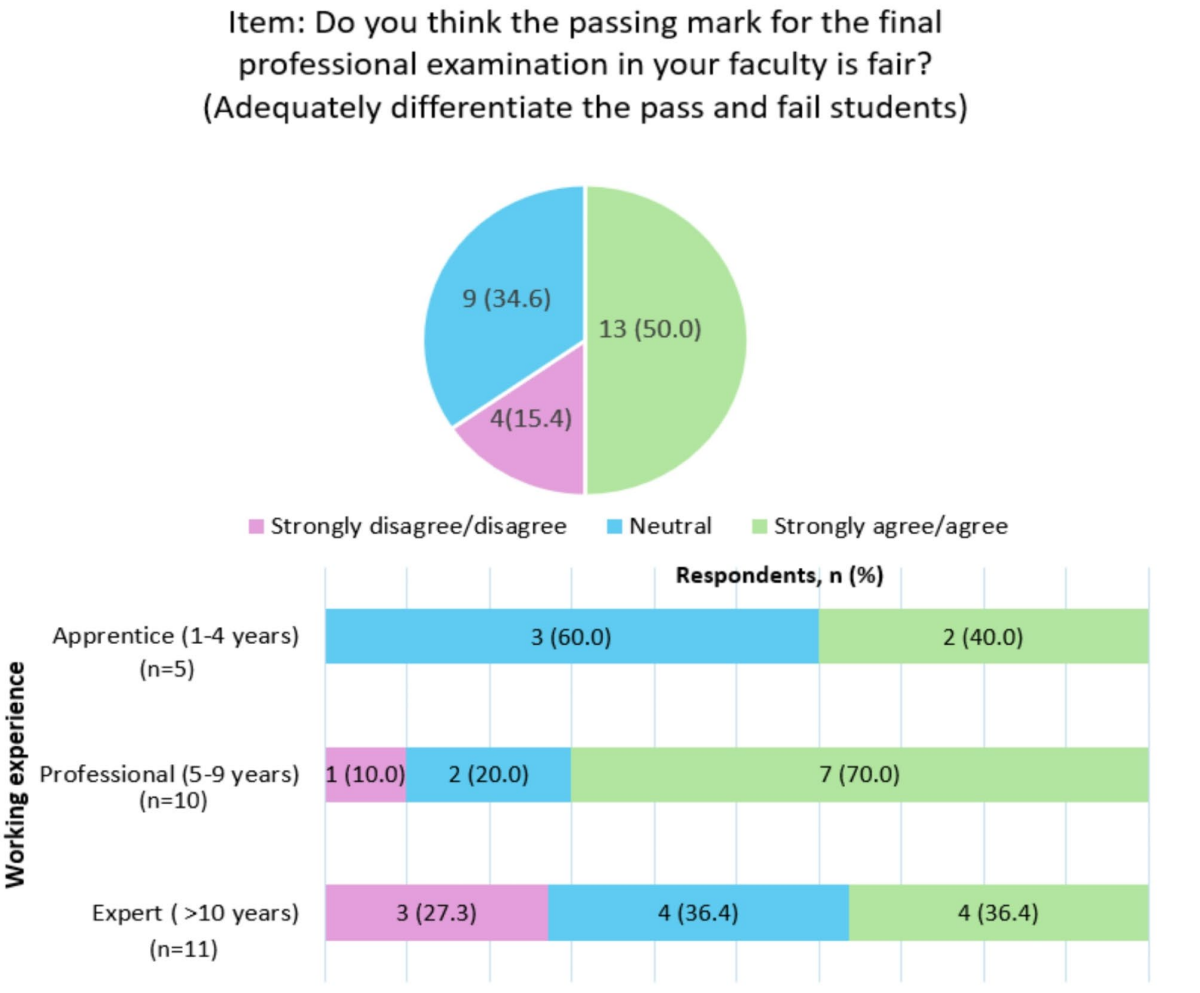
Distribution of responses on the fairness of the passing mark in final examination across different working experience levels in the faculty

**Fig. 4:**
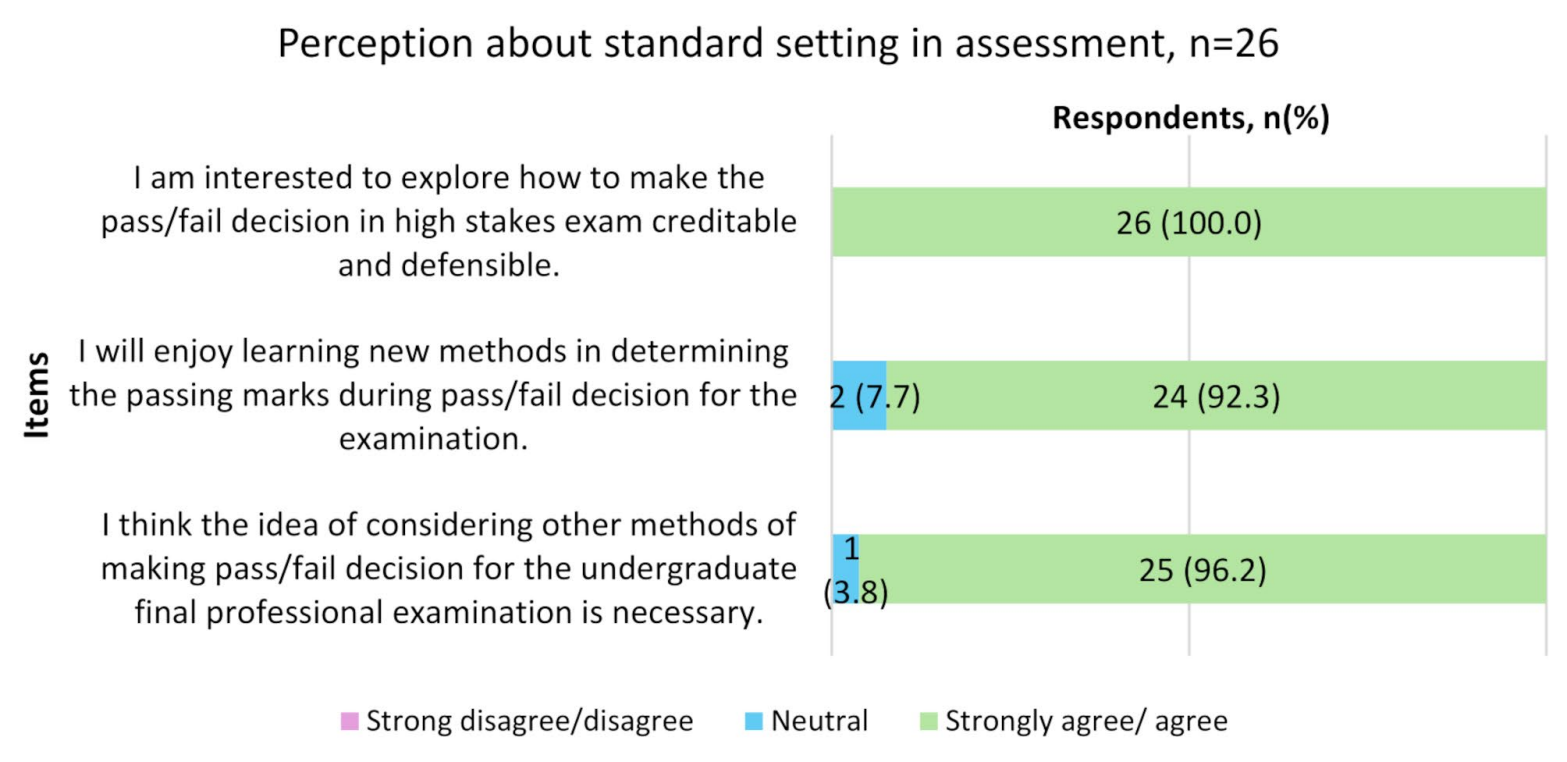
Perception about standard setting in assessment

Figure 3 displays the distribution of responses regarding the fairness of the passing mark in the final examination, based on the Likert scale. For analysis, the ‘strongly disagree’ and ‘disagree’ groups were combined, as well as the ‘strongly agree’ and ‘agree’ groups. The pie chart shows that out of 26 participants, 50.0% of them strongly agree/agree that the passing mark for the final professional examination is fair, 34.6% of them were neutral while 15.4% strongly disagreed/disagreed. The stacked bar chart highlights differences in response distribution across different working experience levels. Among the four respondents who strongly disagreed or disagreed, three belonged to the expert group, whereas those who strongly agreed or agreed (*n* = 13) were predominantly from the professional group (*n* = 7). The chart in Fig. 4 summarises participants’ strong agreement and interest in exploring ways to improve the pass-fail decision-making process, with nearly all respondents favouring learning new or alternative standard setting methods in assessments.

Figure 3 Distribution of responses on the fairness of the passing mark in final examination across different working experience levels in the faculty.

### Qualitative data

The summary findings from the thematic analysis are presented in Table 3. There were four themes each with subthemes identified from initial coding. Quotes from participants are reported to support these themes.

**Table 3:** Identified themes and subthemes related to perceptions on assessment standards and fixed passing marks in undergraduate final examinations

**Theme 1: Trust in the institutional quality assurance processes**
Subthemes: 1. Competency-based framework 2. Composite examination structure 3. Academic governance in place for quality assurance for examination
**Themes 2: Reflections on the passing mark as passing standard**
Subthemes: 1. Institutional policy 2. Scoring reliability can lead to false positives and false negatives 3. Clinical subjects should have stricter passing criteria 4. Passing standard equivalent to minimal competency equivalence
**Theme 3: Potential barriers to standard setting**
Subthemes: 1. Lack of understanding in standard setting leading to informal standard setting practices 2. Resources constraint
**Theme 4: Future faculty development strategies**
Subthemes: 1. Post-hoc assessment analysis 2. Assessment unit and training to enhance assessment practices

### Theme 1: trust in the institutional quality assurance process

**Competency-based framework.** The competency-based assessment implemented by the institution gave the teaching staff confidence that graduates have achieved a minimum mastery of clinical skills and sufficient clinical exposure to be safe dentists.

“*We first evaluate the clinical competency by our competency test throughout the years of study…when they are clinically competent*,* then only they go and sit for their final theory exam*,* which I think if they pass it*,* they are going to be a very competent dentist*” (Participant 11, Apprentice).

“*Those who have not achieved the minimum requirement to sit for the examination*,* they’re not allowed the entry in the examination. So what is the minimum requirement is basically what the clinical exposure*,* as well as the various competency test that they have to sit in. Those safe and competent are the ones who will be allowed to go on*” (Participant 7, Expert).

**Composite examination structure.** The final professional examination marks are derived from multiple sources of assessment across different domains throughout the clinical years. This aggregation of marks provides a longitudinal assessment and mitigates the potential bias associated with a single encounter or assessment.

“*The undergraduate it has always been 50*,* 50*,* that 50% continuous assessment*,* and then 50% from the professional examination itself… Not only based on a knowledge mode. You’ll also have psychomotor mode and affective mode of component. So a student should pass all three components for them… graduating from that programme*” (Participant 5, Expert).

“*One is the continuous assessment*,* which is 50%*,* and another is [end of year] exam*,* which is another 50% [final exam]*,* so they need to fulfil both…We have many tests from year 4 to year 5*,* and also clinical requirements which they need to fulfil… there’s a lot of components for continuous assessment*” (Participant 6, Expert).

*“…every mark that they got it [in final professional exam]*,* the marks is heavily squeezed into a smaller number because of the percentages everything. So the one mark increase in the actual final mark*,* probably 10 marks on the continuous assessment*” (Participant 10, Expert).

**Academic governance in place for quality assurance for examination.** Participants expressed assurance that the assessment employed a constructive alignment approach, utilising an assessment blueprint to ensure tests are aligned with course learning outcomes and matched to appropriate levels of Bloom’s Taxonomy. The presence of assessment committees, comprising both internal and external experts, ensures rigorous review of examination items, processes and outcomes. They reached a consensus in decision-making regarding students’ pass or fail status.

“*We follow the blueprint and we follow the Taxonomy level for the type of questions we set… like we instruct the examiners to set the questions according to this learning outcome to what level*,* so*,* and then the examiners will set the questions accordingly*” (Participant 2, Expert).

*“Rather than actually assessing on our own*,* we also call what we call an independent assessor*,* so this external examiner is also having a similar course in their universit*y” (Participant 4, Expert).

*“The passing or the fail of the student doesn’t actually come from one person*,* it comes from a consensus of the meeting. From the examiners meeting and then go to the department meeting and then after that*,* it goes to the faculty meeting”* (Participant 4, expert).

### Theme 2: reflections on the passing mark as passing standard

**Institutional policy.** Few participants had neutral opinions about the norm in university policy that accepts 50% as the passing mark. They viewed it as an established norm that has been consistently used and rarely questioned in terms of its fairness.

*“I think traditionally*,* people set it at 50%. So that means if you achieve 50% of the mark*,* you are so-called competent and safe enough to work*” (Participant 7, Expert).

“*We do follow the university grading… We have never had any discussion about that*,* because it’s always that way”* (Participant 8, Professional).

**Scoring reliability can lead to false positives and false negatives.** Conversely, participants expressed uncertainty when evaluating students with borderline passing marks between 49% and 51%. Conducting borderline viva voce assessments or reviewing overall performance across all components was aimed at avoiding false negatives. One participant raised concerns about compensatory scoring, where the pass-fail decision was based on a single overall score derived from the candidate’s performance across the entire examination.

“*Looking at a small number between 49 to 51 or 52*,* we have to look at other components to dictate whether this student is worthy to pass or not. Because the range is quite small*,* so is just the matter of luck when we are talking about 49 to 51 or 52*” (Participant 9, Apprentice).

“*…we have the borderline viva so at that stage we tried to differentiate between who should be pass and those who cannot proceed further*” (Participant 1, Expert).

*“…we didn’t set that students must pass every component. So in actual fact*,* student who passed*,* they maybe they fail in certain components*,* say they fail the clinical viva*,* but for the overall marks they pass”* (Participant 2, Expert).

**Clinical subjects should have stricter passing criteria.** Participants perceived that raising the passing mark for theoretical components of clinical subjects is necessary to ensure higher-quality dentists and to prevent incompetent dental students from passing.

*“I think like in terms of writing papers in your writing exam*,* they should at least pass around 60% I guess? 50%*,* I think it’s too low… So that the quality of graduate or dentist that we produce is*,* I think*,* is much better”* (Participant 3, Apprentice).

*“if you have a full mark of 100*,* so which means a passing of 50% means the student can pass by knowing half of the knowledge. Because I think to be a dentist*,* I think you should know more than half of the knowledge or half of the competency”* (Participant 4, Expert).

**Passing standard equivalent to minimal competency equivalence.** Some participants argued that the difficulty level of the exam papers varies from cohort to cohort. They highlighted that the passing mark should be equated to align with the difficulty of the papers and have sufficient discriminatory power to clearly distinguish between pass and fail categories.

“*So if it’s represented by 50%*,* 60%*,* 40%*,* so those numbers are just numbers for us to translate that and to calculate at the end of the day. But is the performance that we are focusing on the achievement of the competency that we are looking at*” (Participant 12, Expert).

“*Of course*,* this is not the ideal kind of thing*,* because*,* the passing mark can differ from year to year if you based on the difficulty index of the question. The score can differ from one batch to another*,* depending on the difficulty of the question*” (Participant 5, Expert).

“*The passing should be tally or equivalent to what we call competency*” (Participant 4, expert).

### Theme 3: potential barriers to standard setting

**Lack of understanding in standard setting leading to informal standard setting practices.** Most participants interviewed had prior experience in examination items writing and developing course assessments. However, many lacked exposure and guidance on testing measurement, leading to varied practices in setting standard based on their own experience.

“*Seriously I don’t know the methods like the proper methods*,* like to set a standard. You know like a standard bar or standard passing mark”* (Participant 3, Apprentice).

*“I would probably put some more basic questions that occupies the 50% and another 50% or less at a much more higher thinking level to pass them”* (Participant 11, Apprentice).

*“Some questions are a bit easy*,* and some questions are a bit more challenging. But as a whole*,* we do not say*,* like*,* okay*,* 20% or 10% of this question of the whole paper has to be like an easier kind of question. And maybe how many percent is intermediate and how many percent is more challenging…it just comes in naturally*,* so meaning that different cohorts may have different percentages of easy medium and difficult questions”* (Participant 7, Expert).

**Resources constraint.** Participants revealed inadequate opportunities for training in educational testing measurement and assessment, which was further compounded by time constraints.

*“… the university where I work does not practice standard setting and I was not trained to do standard setting” (Questionnaire*,* Expert).*

*“Ideally as a person who set the questions should look how the student answered to determine whether the items that we place for the questions are a good ones or not. whether they distractor we put are good distractor or not*,* then we can actually modify from that. But I think most of us are not trained to look at the report.”* (Participant 7, Expert).

*“And it’s usually*,* the big problem is timing*,* I mean*,* and finding the right time to sit down*,* get proper exposure and training. The work and time constraint here. The training is not comprehensive”* (Participant 12, Expert).

### Theme 4: future faculty development strategies

**Post-hoc assessment analysis.** Participants suggested routinely analysing assessment data after examinations to evaluate the effectiveness and reliability of the assessments.

*“Every discipline should evaluate the level of difficulty… we always assume what we give is the right thing and not looking at the student point of view of whether they understand or understood the question or not…. But what we have not done is the after the examination… I think it is also must be made compulsory…to ensure that the questions that we give of good quality*,* and it assess what we want to assess”* (Participant 5, Expert).

*“We have not done the assessment for looking at each question that we give. In terms of the difficulty of the questions…I think we should. Every department or every unit should have that. You know you vet for questions. And then the students answered the questions*,* but they never evaluated whether the questions were suitable for the students or not”* (Participant 6, Expert).

**Assessment unit and training to enhance assessment practices.** Participants expressed that they were positive about quality improvement in the assessment system. The participants expressed the need for better training in assessment, not only in item development but also in evaluating the examination process.

*“I don’t know if 50% is a good mark for passing the students. It triggers a question for myself also. But maybe it’s time for us to look at it. I’m sure that is a standard way of doing it*” (Participant 7, Expert).

*“I suppose we need more people to be trained*,* ah*,* I mean*,* you know*,* to teach us on*,* not just doing questions for exams*,* but also how to evaluate whether it’s suitable or not”* (Participant 6, Expert).

*“You know the questions and keeping and maintaining the bank*,* at least at the undergraduate level*,* not for the postgraduate level. It must be the dedicated examination unit that oversees the running of all the examination”* (Participant 5, Expert).

## Discussion

Different stakeholders in dental education, including students, academics, professional accreditation bodies, and future employers have varying perspectives on the fairness of the passing mark for awarding a dental bachelor’s degree. This mixed-method study explored the experience, practice and perceptions of faculty members in a Malaysian dental school with regards to how the passing mark is decided and how defensible this is.

Our research revealed mixed confidence among participants in using an arbitrary fixed passing mark to make pass-fail decisions for the final professional examination. While half of the participants agreed with the 50% passing mark, the other half remained neutral or disagreed, reflecting uncertainty about its fairness in distinguishing between passing and failing students. Confidence stemmed from a general trust in institutional quality assurance processes, though some participants questioned the reliability of the scoring system in determining the passing standard. Most participants agreed that analysing candidates’ performance data could be useful for setting massing marks. However, limited exposure and training in assessment and educational measurement, as well as resource constraint have restricted the application of post-hoc assessment analysis and standard setting practices. Despite this, participants expressed interest in exploring and learning methods to improve assessment practices and ensure fair passing standards.

The results provide valuable insights for the dental fraternity in non-western countries like Malaysia to evaluate the need for improvements in the examination system. A recent study on assessors from Nigeria’s Postgraduate Medical College surveyed the acceptance of standard setting after its application to five examination diets. Despite 76% believing that the previously used fixed arbitrary passing mark was defensible, 92% supported the implementation of standard setting in college examinations [39]. Reasons quoted by respondents to support standard setting included being evidence-based, not arbitrary, discriminatory, conforming to international best practices, fair, and reproducible. Only one out of 49 opposed its implementation, citing inefficiency. Our study revealed that 50% of respondents trust the fairness of the arbitrary passing marks, 35% were neutral, and 15% disagreed, believing the passing marks do not adequately differentiate between passing and failing students. However, the respondents had no experience in practicing standard setting.

The interview data gathered suggest that participants’ trust in the fixed passing mark is really derived from the fact that institutional rules mean that students must pass minimum clinical competency levels to be eligible to sit the final exams. This means that only students considered to be clinically competent are in a position to pass the exam. For the participants of our study, this safeguard mitigated concerns about the arbitrary nature of the final examination passing mark. This integrated a competency-based education framework is a model widely adopted by institutions internationally [40–43]. Assessment in competency-based education focuses on defined outcomes and measurable competencies through formative or summative assessments that track the students’ performance [44]. This approach shifts from merely counting the number of completed procedures to evaluating learners’ abilities at the ‘does’ level of Miller’s pyramid, ensuring they can perform day-to-day clinical tasks safely and independently based on workplace-based assessments [45, 46]. Based on the interview data, students who do not meet these standards are granted an additional semester to fulfil the requirements and take the final professional examination at a later date. Therefore, they are confident that the graduates have at least achieved minimum clinical competence. The awarding of a dental bachelor’s degree by local schools effectively grants a license to practice, with the MDC requiring graduates to meet minimum clinical experience and competency levels [2]. In addition to this, the engagement of an external examiner for the professional examination, in adherence to both national and international quality assurance guidelines, ensures that the examination processes are transparent and fair. External examiners serve as independent validators who assess whether examination procedures are effectively implemented and compliant with established standards, ensuring that qualifications are awarded impartially [47, 48].

According to the participants, the final score is the composite of multiple testing formats, such as SBA, SAQ, Objective Structured Clinical Examination, oral examinations, portfolios and workplace-based assessments according to the assessment time map and aligned with the purposes of the assessments. The continuous assessments which evaluate students’ overall clinical performance over time, effectively assess professionalism and the affective domain than single-encounter tests. Incorporating multiple assessment points across different competencies and domains aligns with global practices and guidelines, as it enhances the reliability and validity of the evaluation process [49]. One participant raised a concern about compensatory scoring, where poor performance in one component is offset by good performance in another, potentially leading to misclassification of the pass-fail outcome. In compensatory scoring, the pass-fail outcome is determined by a single composite score derived from overall performance across different test formats. In contrast, conjunctive scoring requires candidates to pass each individual component of the examination to pass the overall assessment. In healthcare assessments, educators often strongly believe that passing each test paper, is necessary for a student to pass, regardless of their overall score [14]. However, psychometric analysis has shown that compensatory scoring tends to have higher reliability than conjunctive scoring particularly in terms of decision accuracy and consistency [50, 51]. UKM ensures candidates achieve minimum clinical experience and competencies before the final exam, balancing academic fairness with essential clinical skills through competency-based education and controlled compensation in exams. While compensatory scoring risks compromising patient safety and clinical competence, faculty or policymakers must consider whether all skills or domains are essential or if overall competency is more appropriate for pass-fail decisions.

During the interview, the participants quoted that to reduce the risk of false negatives (failing competent candidates) among those with borderline final scores, a borderline viva voce assessment was conducted. The borderline viva serves as a safety net for false negatives but no efforts were made to address false positives (passing incompetent candidates). Wass and her colleagues recommended that, instead of conducting short viva voce assessments for students with borderline results, efforts should be directed towards increasing test reliability to provide more confidence in pass or fail decisions [52]. No assessment is entirely reliable, and every testing and measurement is subjected to error. Reliability analysis enables educators to calculate the standard error of measurement (SEM), which helps to establish confidence intervals in estimating the true score. Adjusting the pass mark using SEM (+/- 1,2, or 3 SEM) also provides the examinee with “the benefit of doubt” to mitigate the consequences of false positive and false negative decisions [53].

In the interviews, some participants highlighted that the passing standard should reflect the minimum competency level and standards required for graduates to practice safe dentistry. However, there were also perceptions among the participants that higher passing marks are required to provide better quality dentists and thus reduce the risk of false positives. Many faculty members lack formal training in curriculum design, pedagogical theory and assessment development, as most faculty development programmes primarily focus on enhancing teaching and learning skills. Standard setting was a new concept for many academics we interviewed. Due to limited exposure, training, and knowledge in assessment and measurement, post-hoc assessment reports were not routinely analysed [54], making it difficult to ensure the fairness of pass-fail decisions based solely on descriptive statistics. These practices did not align with standard assessment management practices for high-stakes exams like national licensing examinations [19, 23]. The item discrimination index and distractor efficiency are valuable measurements for assessing the quality of the items, whereas item correlation coefficient and Cronbach’s alpha at the item level and scale level respectively are essential for demonstrating the internal consistency and reliability of the testing results [53, 55, 56].

In addition to inadequate exposure to standard setting and time constraints were also identified as barriers to implementing standard setting methods in assessments. Similarly, Rosa et al. (2006) found that excessive workload, budget limitations, and a lack of expertise were among the most commonly reported obstacles to quality assessment. A smaller proportion cited resistance to change, aversion to certain concepts underlying quality management, and general disinterest as additional challenges [57]. The majority of participants demonstrated a positive attitude toward acquiring new knowledge in quality assurance methods for assessment. Understanding their perspectives is essential for assessing the current conditions and evaluating their willingness to accept changes in existing practices.

The passing standard should be justifiable to prevent the passing of incompetent graduates as a result of luck of random error, which could endanger public safety, and to avoid failing competent candidates, thereby protecting their rights and deterring unnecessary stress to candidates and institutions. Collecting the findings from this study to provide evidence-based recommendations for faculty on staff training in development programmes related to assessment and educational measurement. Although dental faculties are typically smaller than medical faculties, establishing a dedicated dental education unit, similar to its medical counterpart, is essential for facilitating and reviewing educational standards and policy guidelines in assessment management, ultimately enhancing the overall quality of the examination process [58]. Efforts must be directed toward policymakers at both the university and national levels to improve standards and guidelines on examination process in dental education.

### Limitations

There were limitations to this study. First, the generalisability of the study is limited as the result of this study is represented by a small sample size in a single dental institution in the local context.

Second, the response rate for the questionnaire was 55%. While suboptimal, it was comparable to the accepted average reported in the literature [59]. Study by Baruch and Holtom (2008) found that the mean response rate from participants in the education and healthcare sectors was 49.0% and 53.8% respectively. They identified several factors contributing to low response rates, including failure to deliver, ‘over-surveying’, time constraints, perceived lack of relevance, inability to return the questionnaire, and company policies prohibiting survey participation. Efforts such as follow-up emails, guaranteed anonymity for the online questionnaire, and incentives for interviews were applied to encourage more recruitment. However, achieving complete anonymity was not feasible in the interview process as the researcher was aware of the participants’ identities.

Third, a criterion sampling method with voluntary selection was applied in this study. Purposive sampling based on diverse backgrounds among the lecturers, such as academic position, years of experience, and discipline, would have been preferable to ensure maximum variation among the participants [60]. However, due to the small sampling frame, there was a potential risk of coercion which could compromise ethical standards. Therefore, participants were not approached based on specific criteria; instead, voluntary participation was encouraged among staff members. We have recruited volunteers from various clinical disciplines and experience levels to participate in the study. We observed higher recruitment among professional and expert groups, likely due to the prevalence of senior lecturers teaching final-year students. We also acknowledged that the voluntary sampling method for both the questionnaire and one-to-one interviews may introduce self-selection bias. The volunteers might not represent the broader sample frame, as they could exhibit greater enthusiasm and motivation than others [61].

Additionally, the main researcher in this study is an insider in the organisation researched, potentially leading to unintentional bias in the data collection and analysis process. The constant reminder of the practice of reflexivity approach throughout the whole process is critical in qualitative research [62]. Researchers vigorously analysed qualitative and quantitative data to reduce reporting risk.

Future research directions may be to gather insights from multiple dental schools, including both public and private institutions providing a more comprehensive and generalisable understanding of the passing mark perceptions for awarding a dental bachelor’s degree. Extending this research to various stakeholders such as students, professional accreditation bodies, and future employers may provide insights into the perceived fairness of arbitrary fixed passing marks and their impact on the quality and outcomes of dental education.

## Conclusion

This study is the first to explore perceptions of the passing standard and fixed passing marks on high-stakes examinations within a dentistry programme among academic staff. The implementation of a new quality assessment system necessitated academic support, and the findings from this research can inform the need for such systems by integrating academics’ views on these matters.

Arbitrary passing marks are common practise in dental education in this region. Our research revealed mixed confidence among participants in using an arbitrary fixed passing mark to make pass-fail decisions for the dental high-stakes examinations. Low level of exposure and knowledge about educational measurement have restricted the application of post-hoc assessment analysis and standard setting practices at the institute. Majority participants were optimistic towards exploring and learning methods to improve assessment practices and ensure fair passing standards. We recommend that policymakers and faculty foster a supportive environment to ensure fair passing marks in examinations.

## Electronic supplementary material

Below is the link to the electronic supplementary material.

Supplementary Material 1

Supplementary Material 2

## Data Availability

The transcripts and datasets collected and/or analysed during this study are not publicly available due to the confidentiality of the data. However, they can be obtained from the first author, Ting Khee Ho upon request.
