# Supplementary material for: Investigating assessment standards and fixed passing marks in dental undergraduate finals: a mixed-methods approach
Source: BMC Med Educ. 2025 Apr 3;25:481. doi: 10.1186/s12909-025-06944-y (PMC11969796; doi:10.1186/s12909-025-06944-y)
Supplement: Supplementary file 1 — Supplementary Material 1 [file 12909_2025_6944_MOESM1_ESM.docx]

**Title: Investigating Assessment Standards and Fixed Passing Marks in Dental Undergraduate Finals in The National University of Malaysia.**

This survey should take less than 15 mins to complete.

**Part 1: Teaching Experience**

1. How many years of experience do you have in teaching FINAL year dental students?

- Years __________________________________________________

2. What is the main discipline in your teaching?

- Dental public health
- Endodontics
- Oral medicine and oral pathology
- Oral Surgery/Oral Maxillofacial Surgery
- Orthodontics
- Paediatric dentistry
- Periodontics
- Prosthodontics
- Restorative dentistry
- Others __________________________________________________

**Part 2: Experience in assessment management and analysing performance data of assessment**

You might have some experience in assessment. Please indicate how much experience, if any, you have in each of the following assessment activities.

**1.** Writing multiple choice question (MCQ)/one best answer (OBA) question

- None
- Yes

If Yes, the number of years involved in the activity

________________________________________________________________

If yes, frequency of the activity

- Once / year
- More than once / year
- Others: __________________________________________________

**2.** Writing multiple short answer /multiple short essay question

- None
- Yes

If yes, the number of years involved in the activity ________________________________________________________________

If yes, frequency of the activity

- Once / year
- More than once / year
- Others: __________________________________________________

**3.** Developing course's assessments (collecting and compiling questions) for your students

- None
- Yes

If yes, the number of years involved in the activity

________________________________________________________________

If yes, frequency of the activity

- Once / year
- More than once / year
- Others: __________________________________________________

**4.** Analysing test-takers’ responses to the choices in MCQ/OBA questions

- None
- Yes

If yes, the number of years involved in the activity

________________________________________________________________

If yes, frequency of the activity

- Once / year
- More than once / year
- Others: __________________________________________________

**5.** Analysing test statistics, such as difficulty index, discriminative index, distractor efficiency in MCQ/OBA questions

- None
- Yes

If yes, the number of years involved in the activity

________________________________________________________________

If yes, frequency of the activity

- Once / year
- More than once / year
- Others: __________________________________________________

**6.** Developing large-scale educational assessment, such as at national level, Professional Qualifying Examination (PQE)

- None
- Yes

If yes, the number of years involved in the activity

________________________________________________________________

If yes, frequency of the activity

- Once / year
- More than once / year
- Others: __________________________________________________

**7.** Setting performance standards which determine passing mark on an assessment

- None
- Yes

If yes, the number of years involved in the activity

________________________________________________________________

If yes, frequency of the activity

- Once / year
- More than once / year
- Others: __________________________________________________

**8.** Have you taken any trainings/courses in assessment, testing, or measurement?

- None
- Yes
- Cannot recall

If yes, please list the training/s or course/s and the duration (hour/day)

________________________________________________________________

**9.** Standard-setting in assessment is a process of establishing the 'minimum pass level' that decides the bar between passing and failing. Below are some of the standard-setting methods that have been used in the assessment. **Which, if any, you have known about it?**

- Nedelsky method
- Angoff method
- Modified Angoff method
- Ebel method
- Bookmark method
- Borderline regression method
- Contrasting groups method
- Hofstee method
- Item Response Theory (IRT)
- Others (plaese specify) __________________________________________________
- I have not known any of these

Display this question if participants choose any of the above standard setting methods, except for ‘I have not known any of these’.

Which, if any, have you experienced in it?

- Nedelsky method
- Angoff method
- Modified Angoff method
- Ebel method
- Bookmark method
- Borderline regression method
- Contrasting groups method
- Hofstee method
- I have no experienced in any of these

**Part 3: Staff’s perception on analysing performance data**

Please rate the following questions according to the scale.

|  | I have no experience in it | Strongly disagree | Disagree | Neutral | Agree | Strongly agree |
| --- | --- | --- | --- | --- | --- | --- |
| 1. Do you think that analysing test-takers’ responses to the choices in MCQ/OBA questions is useful for setting passing marks? |  |  |  |  |  |  |
| 2. Do you think that analysing test statistics, such as difficulty index, discriminative index, distractor efficiency is useful for setting passing marks? |  |  |  |  |  |  |
| 3. Analysing test-takers’ responses to the choices in MCQ/OBA questions will increase my workload. |  |  |  |  |  |  |
| 4. Analysing test statistics, such as difficulty index, discriminative index, distractor efficiency will increase my workload. |  |  |  |  |  |  |
| 5. I enjoy attending the meeting to discuss assessment related subject. |  |  |  |  |  |  |

**Part 4: Perception about setting passing standards in the final professional examination.**

Please rate the following questions according to the scale.

|  | Strongly disagree | Disagree | Neutral | Agree | Strongly agree |
| --- | --- | --- | --- | --- | --- |
| 1. From your experience, do you think the passing mark for the final professional examination in your faculty is fair? (Adequately differentiate the pass and fail students) |  |  |  |  |  |
| Please provide more information to justify your answer.  ________________________________________________________________ | | | | | |
| 2. I am interested to explore how to make the pass/fail decision in high stakes exam creditable and defensible. |  |  |  |  |  |
| 3. I will enjoy learning new methods in determining the passing marks during pass/fail decision for the examination. |  |  |  |  |  |
| 4. I think the idea of considering other methods of making pass/fail decision for the undergraduate final professional examination is necessary. |  |  |  |  |  |

Please leave any additional information that you would like us to know in the space provided below.

________________________________________________________________

________________________________________________________________

This is the end of this survey.

**We would like to invite you to participate in an online one-to-one interview to gain further insight on setting a passing mark in an assessment among faculty members. You do not need to know anything about this topic to take part. We are interested to explore a range of opinions.  Kindly** CLICK HERE **to provide us with your contact details for a follow-up appointment.** Your responses have been recorded. We really appreciate your time and participation in this survey.
