# Supplementary material for: Investigating assessment standards and fixed passing marks in dental undergraduate finals: a mixed-methods approach
Source: BMC Med Educ. 2025 Apr 3;25:481. doi: 10.1186/s12909-025-06944-y (PMC11969796; doi:10.1186/s12909-025-06944-y)
Supplement: Supplementary file 2 — Supplementary Material 2 [file 12909_2025_6944_MOESM2_ESM.docx]

**Virtual one-to-one semi-structured interview guide**

Before I start the interview, I will read this to the participant.

Hi, thank you for accepting the interview. This is the second part of my study which we need to explore the experience, attitudes and perception of setting a passing mark for the Dental Professional Examination among faculty members.

It is important to audio record the interview, so if you decide not to allow audio recording, you will not be able to participate. You can leave the interview at any time if you would like to. The interview will be audio recorded using the ‘record’ function on the software. The audio files will be pseudonymised when storing digitally, transcribed and saved to a secure university computer server. We want all participants to feel comfortable with the recording process and be aware that you are free to stop recording at any time and the information will not be considered in the study. You can also choose to withdraw your interview recording data from the study at any point up until it is used in a publication or research output.

**The listed questions are intended to guide participants in discussing their perceptions of how faculty make pass/fail decisions.**

1. What do you know about the methods that faculty make the pass/fail decision for our undergraduate dental students?
2. What is your view on the ways we make the passing mark or the ways we make the pass/fail decision? (To what extent do you think the passing mark is fair and able to differentiate the pass and fail students?)
3. What are the criteria or components or competencies that you think a student should have when he/she has passed his/her final professional examination?
4. How do you normally set the standard for your questions? (For example, if you choose an easy question or a difficult question.)
5. How far do you think we have done to make sure our passing mark is fair and defensible? (What can we do to ensure we are confident with our pass/fail decision?
6. Is there anything more you would like to add?
